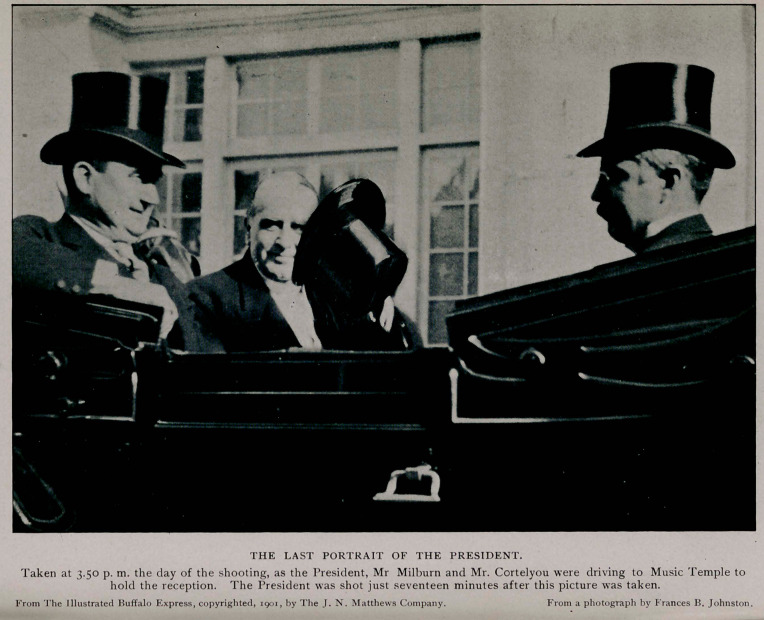# Details of President McKinley’s Case

**Published:** 1901-10

**Authors:** Nelson W. Wilson

**Affiliations:** Buffalo, N. Y., Sanitary Officer, Pan-American Exposition


### Details of President McKinley's Case.

NARRATED BY THE RECORDER AT THE OPERATION.

By NELSON W. WILSON, M. D., Buffalo, N.Y.,
Sanitary Officer, Pan-American Exposition.

WILLIAM McKINLEY, President of the United States, was shot while holding a public reception in the Temple of Music, at the Pan-American Exposition on Friday, September 6, 1901, at seven minutes after four o'clock. His assailant was Leon Czolgosz, a Pole, and an acknowledged anarchist and disciple of Emma Goldman, the most notorious "Red" in the United States.

For full seven days the President lingered, dying at fifteen minutes after two o'clock on the morning of Saturday, September 14, at the residence of Mr. John G. Milburn, to which he had been removed the day of the shooting.

When he was shot the President did not lose consciousness; he saw the crowd fling itself upon his assailant and bear him to the floor; he heard the angry cries for vengeance, and with all the infinite love for mankind and mercy for evil doers which ever characterised his life, this wounded man, grievously hurt as he was, stretched forth his hand as if in benediction and said: "Let no one harm him."

The ambulance clanged its way through the densely packed crowd and tenderly, reverently, the stricken President was lifted from the chair into which he had sunk and was placed on the stretcher. This was lifted into place and the crowd silently and with bared heads parted while the ambulance, surrounded by soldiers and police, whirled away to the hospital.

The dash to the hospital was thrilling and sensational. Mr. T. F. Ellis, who was driving the motor vehicle, handled the steering bar with the utmost skill; no chaffeur however skilful, however expert, ever drove an automobile with more speed and with more wisdom through dangerous places than did Ellis, who is a third-year medical student of the University of Buffalo. The crowd was dense all along the route to the hospital and yet, although the machine was driven at top speed, there were no accidents. Inside the vehicle lay the Chief Magistrate of the United States, carefully attended by Dr. G. McK. Hall and Mr. E. C. Mann, the latter a senior medical student on the staff of the medical department of the Pan-American Hospital.

AT THE HOSPITAL.

Arriving at the hospital the President was taken to the operating room and placed on the table. The doctors of the staff undressed him and ministered to his personal comfort. A hypodermic of morphin was given and almost immediately the telephones began their jingling cry for help. Far and wide through the city went the calls for surgeons.

Dr. Roswell Park, medical director of the exposition, had gone to Niagara Falls to perform an operation. An effort was made to secure the services of Dr. Edward J. Meyer. He, too, was absent from the city. In the meantime Dr. M. D. Mann and Dr. John Parmenter had been sent for and found. Dr. Herman Mynter also was reached, and in a very short time word came that Dr. Park had been communicated with and was on his way from the Falls in a special train.

Dr. Lee, of St. Louis, who was on the grounds at the time of the shooting appeared early and voluntarily assumed charge of the medical department. He was relieved almost immediately by the resident staff. Miss A. M. Walters, superintendent of nurses, displayed excellent judgment in the disposition of the nurses and in the preparations which were immediately begun under her directions for the operation, which her wide experience told her would inevitably follow the arrival of the surgeons. Instruments were sterilised and dressings prepared and when at ten minutes after five Dr. Mann arrived, and with Drs. Mynter and Eugene Wasdin, surgeon of the Marine Hospital Service of the United States, who had reached the hospital a few minutes before, made an examination of the President and decided upon operation, he found everything ready for him. Dr. John Parmenter arrived five minutes later than Dr. Mann and went into consultation with the others.

A careful examination of the wounds showed that the first shot had struck near the middle of the sternum producing simply an abrasion. The second had penetrated the abdomen and was serious. The President's condition was one of shock. Mr. Milburn and the President's secretary, Mr. George B. Cortelyou, with Mr.

SHAKING HANDS WITH THE PRESIDENT.

Taken in the corridor of the Government Building at the Exposition, Sept. 5th.

From The Illustrated Buffalo Express, copyrighted, 1901, by The J. N. Matthews Company.

John N. Scatcherd were called in and informed that immediate operation was a necessity. They told Dr. Mann to do as his wisdom dictated and when the President was informed that an operation was imperative, he said simply: "Gentlemen, I am in your hands."

Again word came that Dr. Park was being rushed to Buffalo. He was on his way. Outside even in the crowd there was anxiety for the coming of the surgeon. On occasions like this a crowd is like a prairie, sun-dried to tinder; a spark of information flashes through it like the tongues of hungry flame. It seemed to one among this vast jam of human beings who were greedily lapping up every thought, every scrap of information from inside, every word, as if they were imbued with the desire to see Dr. Park come to the hospital. How great is a man in the esteem of strangers when, in their hour of anxiety and trouble, they turn to him as a pillar of strength and whisper of him among themselves.

Inside the hospital there was a noiseless bustling to and fro; outside, the waiting crowd, silent and hushed, crushed forward, but needed only a gesture from the police to fall back and sink their voices to whispers. Men with authority forced their way through the jam to the doors of the hospital where they stopped; it was not for them to enter where the forces of science were preparing to do battle with death.

The operating room doors were closed, with secret service agents on guard that none might enter; men who ride rough shod through an every-day life tip-toed their way about; men whose voices ring clear, spoke in hushed whispers with the clutch of sorrow at their throats, for behind those doors in the white room the President lay under the knife of the surgeon; the blood of the Republic was dripping and calm men were searching out the course of an anarchist's bullet, with deft fingers and acute minds.

THE OPERATION.

When the decision to operate immediately had been reached, Dr. Mann assigned their parts to his assistants. He requested Dr. Wasdin to administer the anesthetic, which was begun at twenty minutes after five o'clock, ether being used. Dr. Mynter stood opposite the operator with the Dr. Lee mentioned above; beside Dr. Mann stood Dr. Parmenter as adviser. Dr. Wilson was assigned to take the records of the operation and time. Mr.

Simpson was at the instrument tray; Mr. Mann at the sutures; Miss Catherine Simmons, of Roosevelt Hospital, New York, assisted the anesthetist; Miss M. C. Morris and Miss A. D. Barnes, of St. Luke's, New York, were the sterile nurses; Miss

WHERE THE PRESIDENT WAS SHOT DOWN, IN THE TEMPLE OF MUSIC.

1. Marks the spot where the President stood.

2. The assailant stood here when he fired on the President.

From The Illustrated Buffalo Express, copyrighted, 1901, by The J. N. Matthews Company. From a photograph by Arnold.

Rose Barron, of the Long Island College Hospital, Brooklyn; Miss Mary A. Shannon, of the Cincinnati General Hospital, and Miss L. E. Dorchester, of the Buffalo General Hospital, were detailed as general assistants. Dr. Hall assisted Dr. Zittell in the general care of the hospital during the operation.

In nine minutes the President was under the effects of the anesthetic, and Dr. Mann after preparing the abdomen made a three inch incision, extending through the bullet hole. There was a deep layer of fat which necessitated the lengthening of the incision an inch, when the peritoneum was reached. At the bottom of the incision and in the bullet wound was found a small circular bit of cloth, probably undershirt, which had been carried in by the bullet. On opening the peritoneum the intestines were examined and found to be uninjured. On examination of the stomach a bullet wound was found in its anterior wall. The stomach was drawn up and the wound sutured with a double row of silk sutures. Some stomach contents had escaped from the wound. This was wiped away.

ROSWELL PARK.

From The Illustrated Buffalo Express, copyrighted, 1901, by The J. N. Matthews Co.

The original incision was lengthened two inches so that the posterior wall of the stomach could be examined, and here another bullet wound was found, which was similarly sutured.

In the meantime Dr. Rixey, the President's physician, had arrived from the Milburn house where he had been in attendance on Mrs. McKinley, whose health is extremely delicate. She had but recently passed nigh unto the dark valley and needed the ministrations of a physician almost constantly.

A search was made for the bullet but it could not be found, and as it was in all probability lodged in the deep lumbar muscles it was decided not to make any effort to remove it.

Outside, the crowd, grown denser, hung on every bit of information which trickled from within the white operating room, however meager.

"Is he alive?" an anxious man near the outside door guard would ask. The guard nodded, and the crowd swayed backward and forward with a rustling noise, as the word was passed along in whispers, like a wheat field dipping to a summer breeze.

A little after 6 o'clock an automobile came noiselessly down

MATTHEW D. MANN.

HERMAN MYNTER.

From The Illustrated Buffalo Express, copyrighted, 1901, by The J. N. Matthews Company.

the Mall. There was a sudden switch of the wheels in front of the hospital and it came to a stop. A man in the crowd recognised the passenger as he stepped out and hurried into the hospital.

"That's Dr. Park," he said, and the word was passed along that the surgeon so long expected had arrived. Dr. Park made a record run from Niagara Falls. He was in the midst of an operation when he was notified of the shooting. Rapidly he completed his work after sending word to have an engine ready to take him to Buffalo, and he reached the hospital in two hours after the President had received his wound.

When he entered the operating room he asked what had been done and what had been found, and Dr. Mann told him. The operation was all but completed. All that remained was to flush out the abdominal cavity with salt solution and sew up the abdominal wound. This was done, the abdomen being closed

JOHN PARMENTER.

EUGENE WASDIN.

From The Buffalo Illustrated Express, copyrighted, 1901, by The J. N. Matthews Company.

without drainage. At ten minutes of seven the anesthetic was stopped and the bandages applied. At this time the pulse was 122 and the respiration 32. The President during the operation had been given 1-30 of strychnin and 25 min. of brandy hypodermatically. It was decided after consultation to remove the patient to the residence of Mr. John G. Milburn and a full equipment consisting of bed, bedding and sick room appliances was sent to the house in charge of Miss Simmons and Miss Barnes, who were to take care of the President during the first night. He was placed in the ambulance and in charge of Dr. Park and Dr. Wasdin was moved to his destination.

ANXIETY AND HOPE.

Then came the anxious period of waiting. Bulletins were issued by the President's physicians at frequent intervals. They are published as a part of this article and tell the professional story of the seven days in terse manner. Once or twice will be

P. M. RIXEY.

From a photograph by Clinedinst, Washington.

GEO. B. CORTELYOU.

From a photograph by Frances B. Johnston.

From The Illustrated Buffalo Express, copyrighted, 1901, by The J. N. Matthews Company.

noticed an exultant tone as the condition of the patient improved. There appeared to be no danger until Thursday night. Dr. McBurney, of New York, an eminent surgeon, had arrived on Sunday, and declared the patient would be at his desk in Washington within six weeks. Throughout the land there swelled a feeling of intense relief and gratitude that the life of this good man was to be spared. The surgeons came and went with light steps; they smiled with the confidence the conditions seemed to warrant. Newspapermen, who read events in the eyes of men, heralded the approaching recovery of the President to all the world in positive words; therefore Theodore Roosevelt, Vice-president of the United States, received from the surgeons the assurances that all was well and left for the Adirondacks; the diplomatic corps took trains for their embassies or their summer homes; some of the members of the Cabinet left for their homes; peace and stillness reigned and the joy and thanksgiving of a vast people were unrestrained. Thursday noon, Dr. McBurney also went away. The bulletins said the President had had a piece of toast—some solid food—and that he was on the high road to recovery.

### FEAR AND DESPAIR.

Soon after, late in the afternoon of Thursday, there was a change. A vagueness, an uncertainty, a nameless groping became apparent to the newspaper men on watch. The look in an eye, the lengthening of a facial line, the nervous clicking step, an unwonted activity, told them of a something which had happened. An hour later was the beginning of the end.

The President is not so well; then the President is worse. The President's heart is weak. There has been a collapse. Dr. Charles G. Stockton has been called in consultation. There is grave danger. Strong heart stimulants are being administered.

The world stopped in its work,—a President lay dying. Later came word that the relatives and Cabinet officials had been hurriedly sent for; that couriers were searching the wilderness of the woods for the Vice-president; that Dr. Johnston and Dr. Janeway had been called from their homes in hot haste, to come to Buffalo to the bedside of the man on whom the eyes of the world were turned.

The President does not respond to the stimulants. Unless there is a change soon he cannot live. The doctors have not given up all hope, but there is grave danger. The President has rallied somewhat, but is very weak. The President has suffered another attack of heart failure. The doctors are administering oxygen. The President is dying. The doctors have given up all hope and have stopped the oxygen. The President

From a photograph by Arnold.

THE WOUNDED PRESIDENT BEING TAKEN INTO THE EXPOSITION HOSPITAL.

From The Illustrated Buffalo Express, copyrighted, 1901, by The J. N. Matthews Company.

has said good-by to his wife and death is rapidly approaching.

With tear dimmed eyes, the world awaited, low-bowed with sorrow for the woman, the patient and devoted wife, who had clung so confidingly to the husband who so tenderly watched her when she was battling with the death which now had him in its grasp. She had kissed the white lips; she was holding the hand which had been her protector and her guidance, and he had said good-by. With a world of tenderness and with infinite love he looked at her. It was not a President who was dying. It was a husband bidding a last farewell to a sorely crushed and heart-broken wife; whose last thought was to comfort her in her hour of greatest trial when he whispered:

"God's will be done; not ours."

Then a long and weary wait. The President's eyes closed in unconsciousness and the soul-sick world awaited for the end. Slowly the minutes dragged along and at midnight the President was dying. In the silent chamber the relatives and officials were gathered about the cot where death was reaping the fruits of the blood-red victory of anarchy. The nurses and the hospital corps men of the United States Army moved softly about. Dr. Rixey stood by the side of the form in which barely a breath was left.

THE END.

Outside the wind rustled the leaves of the trees the President loved so well. In the adjoining room slept the wife, mercifully unconscious of the agony of the scene which was being enacted so near her. The respirations became gasps; slower and slower, then halting. Dr. Rixey leaned far forward over the President and raised his hand in warning. A sob broke the awesome silence of the room—a sob which was stifled. The gasping of the President had ceased. Dr. Rixey straightened up, his face drawn with the agony of his suffering. A faint sigh fluttered at the President's lips, and his head sunk into the pillow like that of a man falling into a deep sleep.

An orderly of the hospital corps stepped silently to the foot of the bed and stood rigidly at attention, his eyes to the front.

THE LAST PORTRAIT OF THE PRESIDENT.

Taken at 3.50 p. m. the day of the shooting, as the President, Mr Milburn and Mr. Cortelyou were driving to Music Temple to hold the reception. The President was shot just seventeen minutes after this picture was taken.

From The Illustrated Buffalo Express, copyrighted, 1901, by The J. N. Matthews Company. From a photograph by Frances B. Johnston.

And to the four corners of the world flashed the final bulletin: "The President died at 2.15."

They turned down the lights in the room and left the dead President alone with a white-clad, spectral guard.

THE OFFICIAL BULLETINS.

The official story of the President's case is tersely and scientifically told in these bulletins. The men signing them were Dr. P. M. Rixey, the President's physician; Dr. M. D. Mann, Dr. Roswell Park, Dr. Herman Mynter, Dr. Eugene Wasdin, Dr. Charles McBurney, Dr. Charles G. Stockton and Mr. George B. Cortelyou, secretary to the President. The body of each bulletin is given as it was issued but, for the sake of brevity, the last names of the signers only are printed.

**Friday,** September 6, 7 p.m.—The President was shot about 4 p.m. One bullet struck him in the upper portion of the breast bone, glancing and not penetrating. The second bullet penetrated the abdomen five inches below the left nipple and one and one-half inches to the left of the median line. The abdomen was opened through the line of the bullet wound. It was found that the bullet had penetrated the stomach. The opening in the front wall of the stomach was carefully closed with silk stitches, after which a search was made for a hole in the back wall of the stomach. This was found and also closed in the same way. The further course of the bullet could not be discovered although careful search was made. The abdominal wound was closed without drainage. No injury to the intestines or other abdominal organ was discovered. The patient stood the operation well. Pulse of good quality, rate of 130. Condition at the conclusion of the operation was gratifying. The result cannot be foretold. His condition at present justifies hope of recovery. Signed, George B. Cortelyou, secretary to the President.

10.40 p.m.—The President is rallying satisfactorily and is resting comfortably. Temperature, 100.4°; pulse, 124; respiration, 24. Rixey, Mann, Park, Mynter, Wasdin, Cortelyou.

**Saturday,** September 7, 1 a.m.—The President is free from pain and resting well. Temperature, 100.2; pulse, 120; respiration, 24.

3 a.m.—The President continues to rest well. Temperature, 101.6°; pulse, 110; respiration, 24. Rixey, Cortelyou.

6 a.m.—The President has passed a good night; temperature, 102°; pulse, 110; respiration, 24. Rixey, Park, Cortelyou.

9 a.m.—The President passed a fairly comfortable night and no serious symptoms have developed. Pulse, 146; temperature, 102; respiration, 24. Rixey, Mann, Park, Mynter, Wasdin, Cortelyou.

12 m.—There is no decided change in the President's condition since the last bulletin. Pulse, 136; temperature, 102°; respiration, 28. Rixey, Cortelyou.

3.30 p.m.—The President continues to rest quietly; no change for the worse. Pulse, 140; temperature, 102.2°; respiration, 24. Rixey, Mann, Park, Mynter, Wasdin, Cortelyou.

6.30 p.m.—There is no change for the worse since last bulletin; pulse, 130; temperature, 102.5°; respiration, 29. Rixey, Cortelyou.

9.30 p.m.—Conditions continue much the same. The President responds well to medicine. Pulse, 132; temperature, 102.5°; respiration, 25. All temperatures reported are taken in the rectum. The physicians in attendance wish to say that they are too busily engaged to reply to individual telegrams. Rixey, Park, Mynter, Wasdin, Cortelyou.

**Sunday,** September 8.—The public will be kept fully advised of the actual condition of the President. Each bulletin is carefully and conservatively prepared and is an authoritative statement of the most important features of the case at the hour it is issued. The people are entitled to the facts and shall have them. George B. Cortelyou.

3.20 a.m.—The President has passed a fairly good night; pulse, 122; temperature, 102.4°; respiration, 24. Rixey, Mynter, Cortelyou.

9 a.m.—The President passed a good night and his condition this morning is quite encouraging. His mind is clear and he is resting well; wound dressed at 8.30 and found in a very satisfactory condition. There is no indication of peritonitis. Pulse, 132; temperature, 102.8°; respiration, 24. Rixey, Mann, Park, Mynter, Wasdin, Cortelyou.

12 m.—The improvement in the President's condition has continued since the last bulletin. Pulse, 128; temperature, 101°; respiration, 27. Rixey, Cortelyou.

4 p.m.—The President since the last bulletin has slept quietly—four hours altogether since 9 o'clock. His condition is satisfactory to all physicians present. Pulse, 128; temperature, 101°; respiration, 28. Rixey, Mann, Park, Mynter, Wasdin, McBurney, Cortelyou.

9 p.m.—The President is resting comfortably and there is no special change since last bulletin. Pulse, 130; temperature, 101.6°; respiration, 30. Rixey, Mann, Park, Mynter, Wasdin, McBurney, Cortelyou.

**Monday,** September 9, 6 a.m.—The President passed a somewhat restless night, sleeping fairly well. General condition unchanged. Pulse, 120; temperature, 101°; respiration, 28. Rixey, Mann, Cortelyou.

9.20 a.m.—The President's condition is becoming more and more satisfactory. Untoward incidents are less likely to occur. Pulse, 122; temperature, 100.8°; respiration, 28. Rixey, Mann, Park, Mynter, Wasdin, McBurney, Cortelyou.

3 p.m.—The President's condition steadily improves and he is comfortable, without pain or unfavorable symptoms. Bowel and kidney functions normally performed. Pulse, 113; temperature, 101°; respiration, 26. Rixey, Mann, Park, Mynter, Wasdin, McBurney, Cortelyou.

9.30 p.m.—The President's condition continues favorable. Pulse, 112; temperature, 101°; respiration, 27. Rixey, Mann, Park, Mynter, Wasdin, McBurney, Cortelyou.

**Tuesday,** September 10, 7 a.m.—The President has passed the most comfortable night since the attempt on his life. Pulse, 118; temperature, 100.4°; respiration, 28. Rixey, Park, Cortelyou.

9 a.m.—The President's condition this morning is eminently satisfactory to his physicians. If no complications arise a rapid convalescence may be expected. Pulse, 104; temperature, 99.8°; respiration, 26. This temperature is taken by mouth and should be read about one degree higher by rectum. Rixey, Mann, Park, Mynter, Wasdin, McBurney, Cortelyou.

3.20 p.m.—There is no change since this morning's favorable bulletin. Pulse, 110; temperature, 100°; respiration, 28. Rixey, Mann, Park, Mynter, Wasdin, Cortelyou.

10.30 p.m.—The condition of the President is unchanged in all important particulars. His temperature is 100.6°; pulse, 114; respiration, 28. When the operation was done on Friday last it was noted that the bullet had carried with it a short distance beneath the skin a fragment of the President's coat. This foreign material was, of course, removed, but a slight irritation of the tissues was produced, the evidence of which appeared only tonight. It has been necessary on account of this slight disturbance to remove a few stitches and partially open the skin wound. This incident cannot give rise to other complications, but it is communicated to the public, as the surgeons in attendance wish to make their bulletins entirely frank. In consequence of this separation of the edges of the surface wound the healing of the same will be somewhat delayed. The President is now well enough to begin to take nourishment by the mouth in the form of pure beef juice. Rixey, Mann, Park, Mynter, McBurney, Cortelyou.

**Wednesday,** September 11, 6 a.m.—The President has passed a very comfortable night. Pulse, 120; temperature, 100.2°; respiration, 26. Rixey, Wasdin, Cortelyou.

9 a.m.—The President rested comfortably during the night. Decided benefit has followed the dressing of the wound made last night. His stomach tolerates the beef juice well and it is taken with great satisfaction. His condition this morning is excellent. Pulse, 116; temperature, 100.2°. Rixey, Mann, Park, Mynter, Wasdin, McBurney, Cortelyou.

3.30 p.m.—The President continues to gain and the wound is becoming more healthy. The nourishment taken into the stomach is being gradually increased. Pulse, 120; temperature, 100.2°. Rixey, Mann, Park, Mynter, Wasdin, McBurney, Cortelyou.

10 p.m.—The President's condition continues favorable. Blood count corroborates clinical evidence of the absence of any blood poisoning. He is able to take more nourishment and relish it. Pulse, 120; temperature, 100.4°. Rixey, Mann, Park, Mynter, Wasdin, McBurney, Cortelyou.

**Thursday,** September 12, 6.20 a.m.—The President has had a comfortable night. Pulse, 122; temperature, 100.2°. Rixey, Cortelyou.

9.30 a.m.—The President has spent a quiet and restful night, and has taken much nourishment. He feels better this morning than at any time. He has taken a little solid food this morning and relished it. Pulse, 120; temperature, 100.2°. Rixey, Park, Mynter, Wasdin, Mann, McBurney, Cortelyou.

3 p.m.—The President's condition is very much the same as this morning. His only complaint is of fatigue. He continues to take a sufficient amount of food. Pulse, 126; temperature 100.2°. Rixey, Mann, Park, Mynter, Wasdin, Cortelyou.

8.30 p.m.—The President's condition this evening is not quite so good. His food has not agreed with him and has been stopped. Excretion has not yet been properly established. The kidneys are acting well. His pulse is not satisfactory, but has improved in the last two hours. The wound is doing well. He is resting quietly. Temperature, 100.2°; pulse, 128. Rixey, Mann, Park, Mynter, Wasdin, Stockton, Cortelyou.

12 M.—All unfavorable symptoms in the President's condition have improved since the last bulletin. Pulse, 120; temperature, 100.2°. Rixey, Wasdin, Stockton, Cortelyou.

**Friday,** September 13, 2.50 a.m.—The President's condition is very serious and gives rise to the gravest apprehension. His bowels have moved well, but his heart does not respond properly to stimulation. He is conscious. The skin is warm, and the pulse small, regular, easily compressible, 126; respiration, 30; temperature, 100°. Rixey, Mann, Park, Mynter, Wasdin, Stockton, Cortelyou.

9 a.m.—The President's condition has somewhat improved during the past few hours. There is a better response to stimulation. He is conscious and free from pain. Pulse, 128; temperature, 99.8°. Rixey, Mann, Park, Mynter, Wasdin, Stockton, Cortelyou.

2.30 p.m.—The President has more than held his own since morning and his condition justifies the expectation of further improvement. He is better than yesterday at this time. Pulse, 123; temperature, 99.4°. Rixey, Mann, Mynter, Wasdin, Stockton, Cortelyou.

4 p.m.—The President's physicians report that he is only slightly improved since the last bulletin. The pulse and temperature remain the same as at that hour. Cortelyou.

5.35 p.m.—The President's physicians report that his condition is grave at this hour. He is suffering from extreme prostration. Oxygen is being given. He responds to stimulation but poorly. Pulse, 125; respiration, 40. Cortelyou.

6.30 p.m.—The President's physicians report that his condition is most serious in spite of vigorous stimulation. The depression continues and is profound. Unless it can be relieved the end is only a question of time. Cortelyou.

Here the official bulletins ceased. At short intervals, however, word was sent to the newspaper men in camp opposite Milburn house, as to the downward progress of the President toward the dark valley. We append the two most significant of these.

9.30 p.m.—The President is dying.

**Saturday,** September 14, 2.15 a.m.—The President is dead.

THE AUTOPSY REPORT.

The autopsy was performed by Dr. H. R. Gaylord, and Dr. H. G. Matzinger, of the Buffalo State Pathological Laboratory, on Saturday, the day of death. It was apparently an exhaustive examination occupying several hours. The official report is as follows:

The bullet which struck over the breast bone did not pass through the skin and did little harm.

The other bullet passed through both walls of the stomach near its lower border. Both holes were found to be perfectly closed by the stitches, but the tissue around each hole had become gangrenous. After passing through the stomach the bullet passed into the back walls of the abdomen hitting and tearing the upper end of the kidney. This portion of the bullet track was also gangrenous, the gangrene involving the pancreas.

The bullet has not yet been found. There was no sign of peritonitis or disease of other organs. The heart walls were very thin. There was no evidence of any attempt at repair on the part of nature and death resulted from the gangrene which affected the stomach around the bullet wounds as well as the tissues around the further course of the bullet. Death was unavoidable by any surgical or medical treatment and was the direct result of the bullet wound.

Signed, Harvey R. Gaylord, Herman G. Matzinger, P. M. Rixey, Matthew D. Mann, Herman Mynter, Roswell Park, Eugene Wasdin, Charles G. Stockton, Edward G. Janeway, W. W. Johnston, W. P. Kendall, Surgeon, U. S. Army, Charles Cary, Edward L. Munson, Assistant Surgeon, U. S. Army, Hermanus L. Baer.

Sunday the President's body lay in state at the City Hall from noon until late at night and it is estimated that over 100,000 people gazed at the marble-like features of the dead ruler.

Monday morning the funeral train left Buffalo for Washington.

153 Seventh Street.

---

Reports having been circulated and published to the effect that there was serious disagreement among the President's physicians, a meeting of the staff was held at the residence of Mr. Ansley Wilcox, Tuesday evening, Sept. 17, 1901, to consider the subject. As a result the following statement was issued:

The undersigned, surgeons and physicians who were in attendance on the late President McKinley, have had their attention called to certain sensational statements recently published in the daily papers and particularly in one New York paper, indicating dissension and mutual recrimination among them.

We desire to say to the press and the public, once for all, that every such publication and all alleged interviews with any of us containing criticism of one another or of any of our associates, are false and are nothing but scandal mongering.

We say again that there was never a serious disagreement among the professional attendants as to any of the symptoms or as to treatment of the case, or as to the bulletins which were issued. A very unusual harmony of opinion and of action prevailed all through the case.

The unfortunate result could not have been foreseen before the unfavorable symptoms declared themselves late on the sixth day and could not have been prevented by any human agency.

Pending the completion and publication of the official reports of the post-mortem examiners and of the attending staff we shall refuse to make any further statements for publication, and alleged interviews with any of us may be known to be fictitious.

Matthew D. Mann, Roswell Park, Herman Mynter, Eugene Wasdin, Charles G. Stockton.

## Figures and Tables

**Figure f1:**
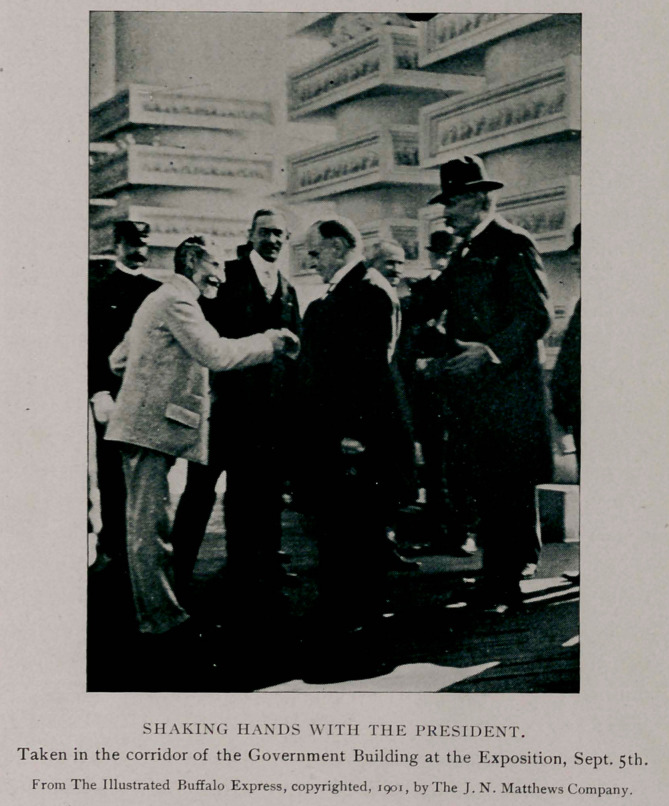


**Figure f2:**
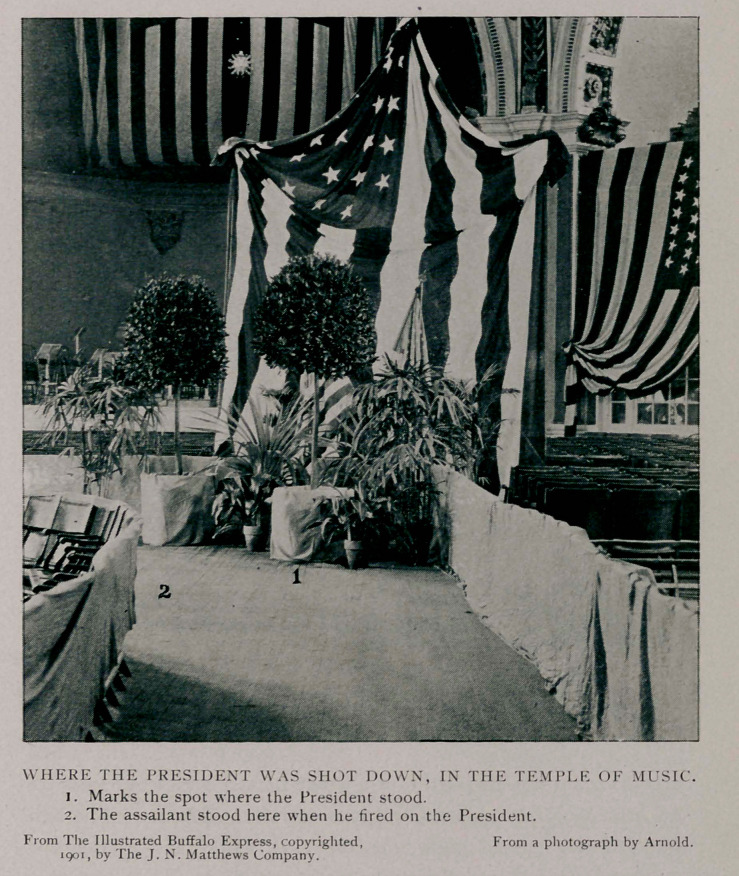


**Figure f3:**
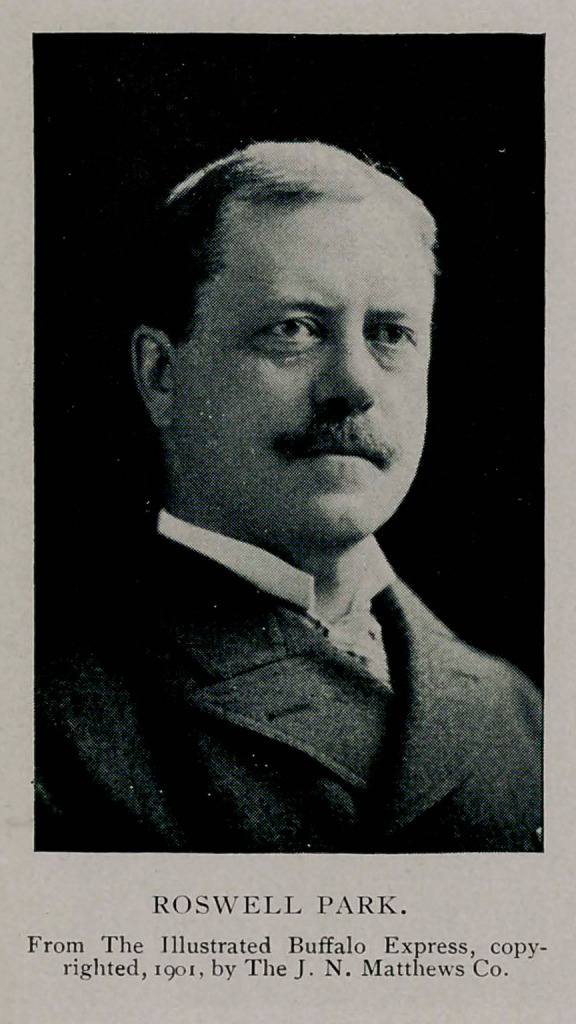


**Figure f4:**
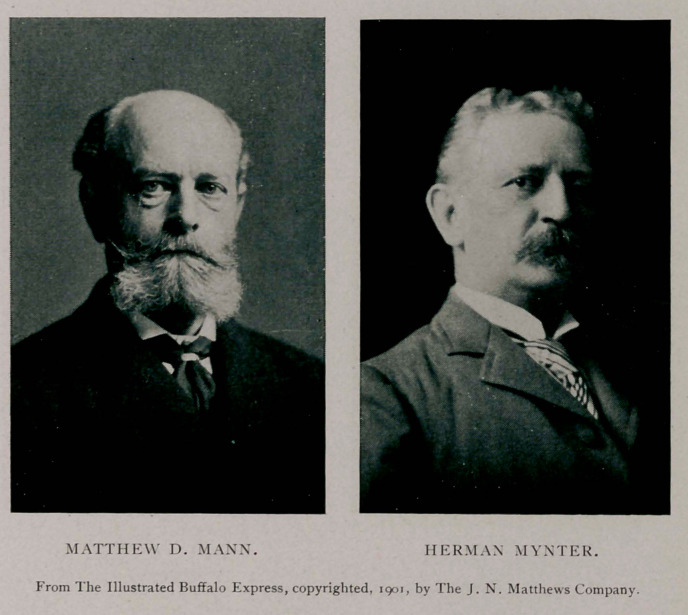


**Figure f5:**
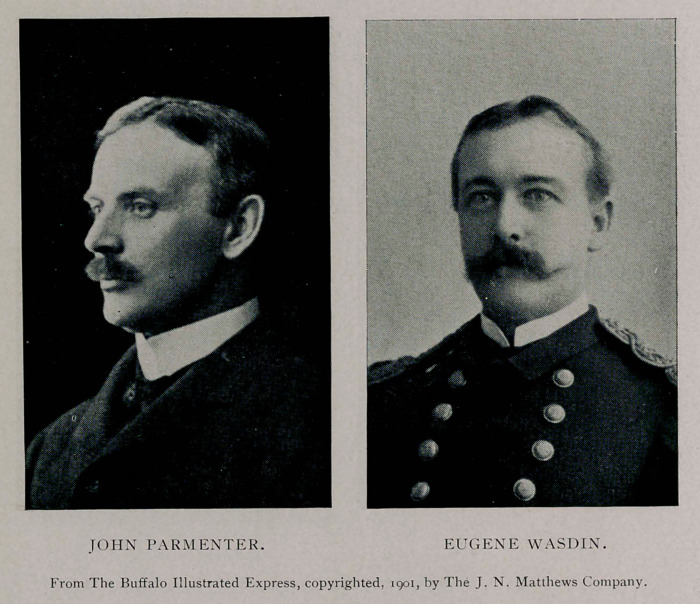


**Figure f6:**
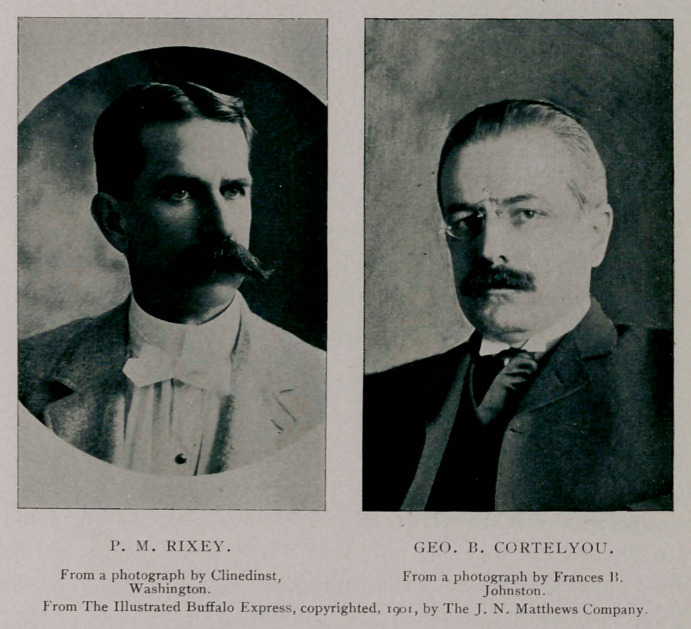


**Figure f7:**
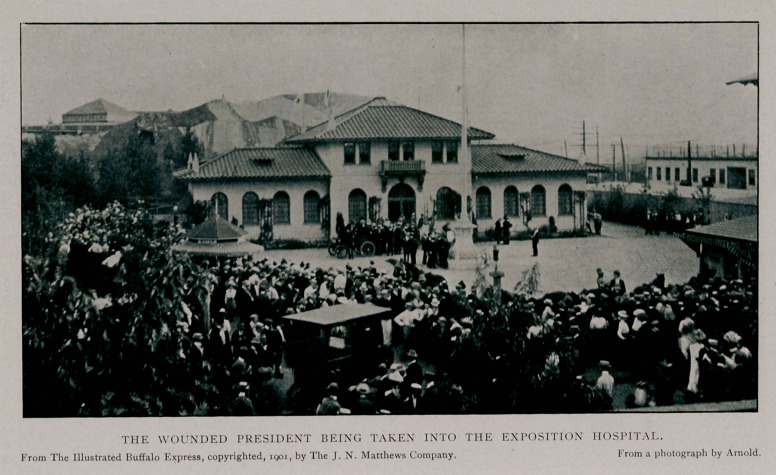


**Figure f8:**